# Characteristics and recovery methods of studies falsely excluded during literature screening—a systematic review

**DOI:** 10.1186/s13643-022-02109-w

**Published:** 2022-11-09

**Authors:** Lisa Affengruber, Andreea Dobrescu, Emma Persad, Irma Klerings, Gernot Wagner, Isolde Sommer, Gerald Gartlehner

**Affiliations:** 1 Department for Evidence-Based Medicine and Evaluation, Cochrane Austria, Danube University Krems, Dr. Karl Dorrek Strasse 30, 3500 Krems, Austria; 2 Department of Family Medicine, Care and Public Health Research Institute (CAPHRI), Maastricht University, Peter Debyeplein 1, 6229 HA Maastricht, The Netherlands; 3 RTI International, 3040 Cornwallis Road, PO Box 12194, Research Triangle Park, North Carolina 27709-2194 USA

**Keywords:** Single screening, Falsely excluded studies, Systematic review, Rapid review

## Abstract

**Background:**

Due to the growing need to provide evidence syntheses under time constraints, researchers have begun focusing on the exploration of rapid review methods, which often employ single-reviewer literature screening. However, single-reviewer screening misses, on average, 13% of relevant studies, compared to 3% with dual-reviewer screening. Little guidance exists regarding methods to recover studies falsely excluded during literature screening. Likewise, it is unclear whether specific study characteristics can predict an increased risk of false exclusion. This systematic review aimed to identify supplementary search methods that can be used to recover studies falsely excluded during literature screening. Moreover, it strove to identify study-level predictors that indicate an elevated risk of false exclusions of studies during literature screening.

**Methods:**

We performed literature searches for eligible studies in MEDLINE, Science Citation Index Expanded, Social Sciences Citation Index, Current Contents Connect, Embase, Epistemonikos.org, and Information Science & Technology Abstracts from 1999 to June 23, 2020. We searched for gray literature, checked reference lists, and conducted hand searches in two relevant journals and similar article searches current to January 28, 2021. Two investigators independently screened the literature; one investigator performed the data extraction, and a second investigator checked for correctness and completeness. Two reviewers assessed the risk of bias of eligible studies. We synthesized the results narratively.

**Results:**

Three method studies, two with a case-study design and one with a case-series design, met the inclusion criteria. One study reported that all falsely excluded publications (8%) could be recovered through reference list checking compared to other supplementary search methods. No included methods study analyzed the impact of recovered studies on conclusions or meta-analyses. Two studies reported that up to 8% of studies were falsely excluded due to uninformative titles and abstracts, and one study showed that 11% of non-English studies were falsely excluded.

**Conclusions:**

Due to the limited evidence based on two case studies and one case series, we can draw no firm conclusion about the most reliable and most valid method to recover studies falsely excluded during literature screening or about the characteristics that might predict a higher risk of false exclusion.

**Systematic review registration:**

https://osf.io/v2pjr/

**Supplementary Information:**

The online version contains supplementary material available at 10.1186/s13643-022-02109-w.

## Background

Screening of titles, abstracts, and full-text publications to identify potentially eligible studies is an essential methodological element of any evidence synthesis. To reduce the risk of erroneously excluding relevant studies during literature screening, most international evidence synthesis organizations recommend dual-reviewer screening, that is, having two persons independently screen titles, abstracts, and full texts [1–3].

Dual-reviewer screening, however, is time-consuming. Due to the growing need to provide evidence syntheses under time constraints, researchers have begun focusing on the exploration of rapid review methods, which streamline the methodological steps of the systematic review process to provide answers more quickly. Rapid reviews often use single-reviewer screening, whereby each record is screened by only a single investigator. Single-reviewer screening reduces screening time by approximately 60% when compared to dual-reviewer screening [4]. The downside of single-reviewer screening, however, is that it is prone to falsely excluding relevant studies. In a crowd-based randomized controlled trial. Gartlehner et al. reported that single-reviewer abstract screening missed on average 13% (sensitivity: 86.6%; 95% confidence interval [CI], 80.6 to 91.2%) of relevant studies [5]. By comparison, dual-reviewer screening missed an average of 3% (sensitivity: 97.5%; 95% CI, 95.1 to 98.8%) of relevant studies [5]. In a recent systematic review by Waffenschmidt et al. on single- versus dual-reviewer screening, the median proportion of relevant but missed studies with single-reviewer screening was 5% (range 0 to 58%) [6].

Consequently, methods to mitigate the risk of erroneous exclusions of relevant studies and to recover falsely excluded studies during the evidence synthesis process are critical to ensure the validity of rapid review results. Table 1 lists several methods that could be used in an attempt to recover falsely excluded studies [7, 8]. These methods are based on supplementary literature searches that can identify studies with a high likelihood of being relevant to the topic of interest. For example, commonly employed methods include reference list checking of the included studies or of other systematic reviews or using the “similar articles” function in electronic databases. The principle behind these supplementary searches is that investigators can reconsider the inclusion or exclusion of articles with a high likelihood of being relevant. In some cases, they might identify studies that investigators falsely excluded during single-reviewer literature screening. However, to date, only half of the published rapid reviews conducted reference list checking of the eligible studies [9].

**Table 1:** Definitions of commonly used methods that could recover falsely excluded studies

Reference list checking (backward citation tracking)	Checking the reference lists of the included studies and any relevant systematic reviews identified [7, 8]
Similarity searches (i.e., related articles)	Using a key article to identify additional relevant articles by using a “similar articles” option available in some databases and search engines (e.g., PubMed, Google Scholar) [7, 8]
Forward citation tracking of included studies	Using citation indexes for forward citation searching based on a key article [8]
Academic search engines	Keyword searches using an academic search engine (e.g., Google Scholar) [8]
Contacting experts/researchers/companies/other stakeholders	Contacting individuals and organizations for information about relevant studies [8]

Additionally, it would be of interest to researchers to know whether certain types of studies or publications have a higher risk of being falsely excluded than others [10]. For example, the publication year could play a role, as abstract reporting standards have changed over time. Older studies might be falsely excluded more frequently during abstract screening since information now considered relevant for an abstract might not have been deemed as important at the time of publication. As another example, one explanation for the false exclusion of studies in the systematic review by Waffenschmidt et al. was that the research question was too vague and largely depended on the interpretation of the reviewer [6].

The aim of this study was to systematically assess which methods have been used to recover studies falsely excluded during literature screening. Additionally, we aimed to identify the potential predictors and characteristics of falsely excluded studies.

## Methods

The aim of this systematic review was to address the following key questions (KQs):

- KQ 1: How effective are different methods in recovering studies falsely excluded during literature screening?
- KQ 2a: What are the characteristics of studies that have been falsely excluded during literature screening?
- KQ 2b: Can predictors help identify studies that are at a high risk of being falsely excluded?

This systematic review was conducted according to Cochrane methods [11]. We followed the Preferred Reporting Items for Systematic Review and Meta-Analysis (PRISMA) 2020 statement [12]. The PRISMA checklist can be seen in Additional file 1. We registered our study protocol “Falsely excluded studies in the literature screening process—a systematic review” at https://osf.io (https://osf.io/5zdpb/). Because we could not identify studies that formally assessed predictors of the false exclusion of records during literature screening, we amended the protocol on June 11, 2021. We expanded the inclusion criteria to also include the study characteristics of falsely excluded studies, even if these characteristics were not formally assessed in a predictive model.

### Eligibility criteria

The a priori–defined eligibility criteria are listed in Table 2 and described in more detail below.

**Table 2:** Inclusion and exclusion criteria

	Inclusion	Exclusion
KQ 1: Methods to recover falsely excluded studies	**•** Reference list checking of included studies **•** Similarity searches (i.e., related articles) **•** Forward citation tracking of included studies using citation indexes **•** Google Scholar search **•** Contacting experts/researchers/companies/other stakeholders	Other methods
KQ 2: Potential predictors of false exclusion	**•** Study design **•** Main objective **•** Sample size **•** Country of conduct **•** Year of publication **•** Structure and content of abstract (e.g., only title available, no abstract, uninformative abstract) **•** Language of publication **•** Risk of bias **•** Database indexing of studies (e.g., PubMed listing) **•** Publication type **•** Journal in which the study is published **•** Impact factor of journal in which the study is published **•** Others	
Outcomes	**•** Proportion of falsely excluded studies that could be recovered (KQ 1) **•** Recall, precision, numbers needed to read (NNR) of supplementary searches (KQ 1) **•** Impact of recovered studies on meta-analysis results and conclusions (KQ 1) **•** Falsely excluded studies by characteristics (KQ 2) **•** Falsely excluded studies by predictors (KQ 2)	Other outcomes
Study design	**•** Systematic reviews (KQ 1, 2) **•** Randomized/nonrandomized trials (KQ 1, 2) **•** Prospective and retrospective, controlled and uncontrolled observational studies (KQ 1, 2)	Nonempirical publications (e.g., editorials, letters)
Date of search	Published 1999 or later	1998 and earlier
Publication language	No restrictions	No restrictions

*Abbreviations*: *KQ* key question, *NNR* numbers needed to read

We searched for studies assessing the use of supplementary search methods (e.g., forward citation tracking, reference list checking, and web searching) to recover studies falsely excluded during literature screening. These supplementary search methods are defined in Table 1.

Additionally, we searched for studies focusing on the predictors and characteristics of falsely excluded studies, such as those based on study design or publication type. Based on internal discussion and consensus among co-authors, we generated a list of potential predictors of false exclusion. This list was not exhaustive, and any other predictor not named on the list would have been eligible. For detailed eligibility criteria, see Table 2.

### Information sources

An experienced information specialist performed searches for eligible studies in MEDLINE (Ovid), Science Citation Index Expanded, Social Sciences Citation Index, Current Contents Connect (all via Web of Science), Embase (Elsevier), Epistemonikos.org, and Information Science & Technology Abstracts (Ebsco) from 1999 to June 23, 2020. We first developed a search strategy for Ovid MEDLINE and then amended it to fit other electronic databases. We considered publications in all languages. According to the peer review of the electronic search strategy (PRESS) statement [12], the electronic Ovid MEDLINE search strategy was peer-reviewed by another information specialist. See Additional file 2 for the database search strategies.

In addition, we searched for gray literature (i.e., unpublished studies) relevant to this systematic review. Potential sources of gray literature included the Open Science Framework (www.osf.io), websites of known organizations that produce rapid reviews (e.g., Canadian Agency for Drugs and Technologies in Health [CADTH]) based on the CADTH Gray Matters Checklist [13], and dissertation databases (e.g., Digital Access to Research Theses [DART]-Europe). Furthermore, we searched for Cochrane Colloquium abstracts of oral, poster, and workshop presentations and Health Technology Assessment international (HTAi) meeting abstracts.

We manually searched the reference lists of background articles on this topic for any relevant citations that our electronic searches might have missed. Additionally, we hand searched journals that regularly publish methods studies, such as *Systematic Reviews* and *Research Synthesis Methods*. If our search retrieved conference abstracts of studies that might have fulfilled our inclusion criteria, we manually searched for further information about these studies (e.g., publications, entries in trial registries, etc.). Additionally, an information specialist conducted similar articles searches for identified key articles in PubMed and Google Scholar and forward citation tracking using Scopus up to January 28, 2021. The search results for the similar article searches are ranked by “similarity” to the key article; the top 20 articles are those categorized as the most similar according to the search algorithm. We exported the top 20 articles and assessed them according to our eligibility criteria. See Additional file 3 for the similar articles searches and forward citation tracking.

### Study records

#### Data management

Identified citations were stored in an EndNote® X8.2 bibliographic database (Thomson Reuters, New York, NY). All results of the abstract and full-text review, including the exclusion reasons during the full-text review, were recorded in the EndNote database. PDF files of all full-text articles were stored on a server accessible to all members of the review team.

#### Selection process

Deduplication of the search results was carried out with EndNote® X8.2 (Thomson Reuters, New York, NY). We developed and pilot-tested abstract and full-text review forms that reflected our inclusion and exclusion criteria. Two independent reviewers screened abstracts and full-text articles in Covidence (www.covidence.org) and evaluated their eligibility for inclusion. Any discrepancies were resolved through discussion or consultation with a third reviewer. A total of 50 abstracts were piloted by all reviewers to resolve discrepancies and to test the abstract review form. The full-text review form was piloted with five full-text articles.

#### Data collection process

We designed and pilot-tested a structured data abstraction form. The data were extracted by one reviewer and checked for completeness and accuracy by a second investigator. The data extraction process was piloted with five studies.

### Data items

For studies that met our inclusion criteria, we extracted the following study characteristics and outcomes:

- Study characteristics: author, year of publication, aims, study design, sample size (e.g., number of studies analyzed), number of reviewers involved
- Characteristics of methods/information sources used to recover falsely excluded studies (for KQ 1)
- Characteristics of falsely excluded studies/publications: study design, content of the abstract, language of publication (for KQ 2)
- Outcomes: proportion of falsely excluded studies/publications that could be recovered, impact of recovered studies on meta-analysis results and/or conclusions, proportion of falsely excluded studies/publications by characteristic or predictor

### Risk of bias assessment

For methods studies with a case-study design, we adapted the Joanna Briggs Institute Critical Appraisal Checklist for Case Reports, and for method studies with a case-series design, the Joanna Briggs Institute Critical Appraisal Checklist for Case Series [14].

### Data synthesis

We summarized the results narratively and grouped them by outcomes of interest. We did not identify enough studies with a similar design to be able to conduct meta-analyses.

## Results

**Fig. 1:**
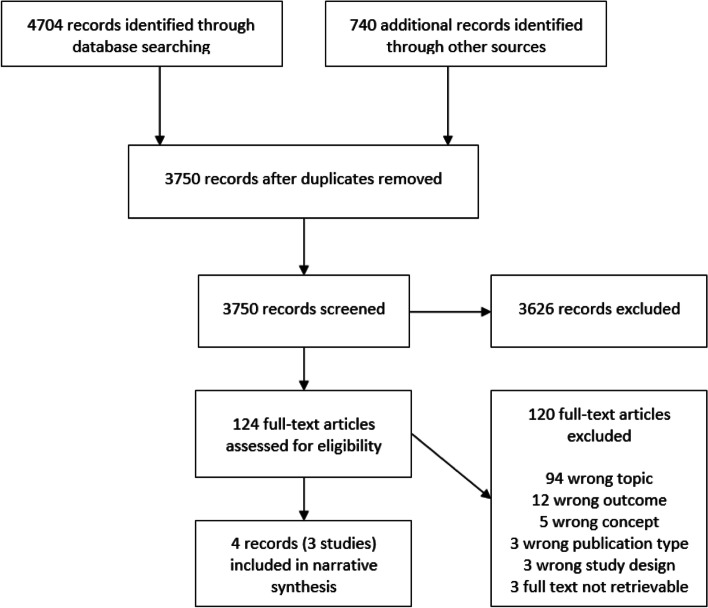
Flow diagram

The literature searches identified 3750 deduplicated unique records, of which 124 full texts were assessed for eligibility. Three studies published in four publications met our inclusion criteria [10, 15–17]. One study reported on KQ 1, all three included studies considered KQ 2a, and no study was identified for KQ 2b. Figure 1 depicts the record review flow. Additional file 4 lists the studies excluded at the full-text level and the reasons for exclusion.

### Characteristics of the included studies

We included three studies (published in four articles), of which two were method studies with a case-study design [10, 16, 17], and one was a methods study with a case series design [15]. We rated the risk of bias of two studies as high [15, 17] and of one study as low [16]. Additional file 5 presents the detailed risk of bias ratings. The aim of the three studies was mainly to assess different methods to accelerate the literature screening process, but they also reported the study characteristics of falsely excluded studies and recovery methods. Rathbone et al. [15] and Feehan et al. [10, 17] mentioned uninformative abstract content details as a study characteristic of the falsely excluded studies. The study by Busse et al. [16] reported falsely excluded studies by publication language, comparing English-speaking reviewers with native-speaking reviewers. The study by Feehan et al. additionally reported their methods to recover those studies falsely excluded during literature screening [10, 17].

For the study by Feehan et al. [17], we identified a companion publication. This conference abstract by Beck et al. [18] was mentioned in a Cochrane review [10] on “Checking reference lists to find additional studies for systematic reviews.” All studies added relevant information for evidence synthesis. Table 3 presents the characteristics of the included studies.

**Table 3:** Characteristics and results of the included studies

Author, year	Key question	Study design	Risk of bias	Aim	N of analyzed SRs, studies, publications	Abstract and/or full-text screening	N of reviewers involved	Proportion of falsely excluded studies/publications by characteristic		Proportion of falsely excluded studies that could be recovered
Busse 2014 [16]	KQ 2	Methods study with a case-study design	Low	To explore the extent to which English-speaking reviewers can differentiate eligible from ineligible foreign-language articles in a systematic review of all treatments for fibromyalgia	1 SR, 53 studies	Full-text screening	16	Single screening: NR	Dual screening: *Non-English language:* Overall: 6/53 (11.3%) German: 2/16 (12.5%) French: 1/4 (25.0%) Turkish: 1/9 (11.1%) Chinese: 1/7 (14.3%) Korean: 1/1 (100%) Italian: 0/5 (0%) Spanish: 0/5 (0%) Portuguese: 0/3 (0%) Dutch: 0/1 (0%) Russian: 0/1 (0%) Polish: 0/1 (0%)	NR
Feehan 2011 [10, 17]	KQ 1, KQ 2	Methods study with a case-study design	High	To track eligible articles identified by checking references lists and to determine which of those had also been found through database searches, but had been screened out by the review authors	1 SR, 119 studies, 134 publications	Abstract screening	8	Single screening: NR	Dual screening: *Abstract content details:* 11/134 publications (8.2%) due to uninformative titles and abstracts of the bibliographic record	11/11 publications (100%) through reference list checking 0/11 publications through contacting key authors, reference list checking, forward citation tracking
Rathbone 2017 [15]	KQ 2	Methods study with a case-series design	High	To evaluate the feasibility of PICo-based title-only screening for scoping searches and rapid reviews by measuring the reduction in screening effort and the maintenance of recall of relevant records	10 SRs, 211 studies	Abstract screening	5	Single screening: *Abstract content details:* 1/211 (0.005%) due to an uninformative title	Dual screening: NR	NR

*Abbreviations: KQ* key question, *N* number, *NA* not applicable, *NR* not reported, *PICo* Participants, Intervention, and Comparator, but not the Outcome, *SR* systematic review

Three methods studies [10, 15–17] assessed studies falsely excluded during abstract screening; one study assessed only full-text screening [16]. The three studies [10, 15–17] assessed one to ten systematic reviews including 53 to 211 studies and involved five to 16 reviewers in their literature screening process. The results for KQs 1 and 2 are described in detail in the following sections and in Table 3.

### Methods to recover studies falsely excluded during literature screening (KQ 1)

One methods study with a case study design [10, 17] conducted a scoping review on an orthopedic topic and analyzed all references included in their review. We rated this study as having a high risk of bias due to it missing conclusions and clear descriptions of the characteristics of falsely excluded studies. The authors found that 11 of 134 eligible studies had been initially found by database searches but had been erroneously excluded during dual screening of the titles and abstracts. The investigators recovered all 11 falsely excluded references (100%) through reference list checking of the included studies [10, 17]. The investigators also performed other supplementary search methods, such as contacting key authors, reference list checking, forward citation tracking, and hand searches of online journal websites, but these methods did not recover any falsely excluded references. The study did not analyze the impact of recovered studies on conclusions or meta-analyses.

### Characteristics or predictors of falsely excluded studies (KQ 2)

All three studies explored the characteristics of studies falsely excluded during literature screening. We could not identify studies that formally assessed whether specific characteristics could predict an increased risk of a study being falsely excluded.

The characteristics mentioned in the studies were uninformative abstract content details and a non-English publication language (Table 3). The following sections summarize these findings in more detail.

#### Abstract content details

Two methods studies, one with a case-study design and one with a case-series design, mentioned uninformative abstract content details as a characteristic for false exclusion [10, 15, 17]. We rated these two methods studies as having a high risk of bias mainly due to them analyzing a convenience case or case series and missing reports of the eligibility criteria and characteristics of the included cases. Feehan et al. [10, 17] documented false exclusions during the production of a scoping review including 134 publications. Eleven of the 134 publications (8%) were falsely excluded due to uninformative titles and abstracts [10, 17]. Rathbone et al*.* [15] assessed a convenience sample of 10 systematic review datasets including 211 eligible studies, derived from the literature searches of completed systematic reviews, to test Participants, Intervention, and Comparator, but not the Outcome (PICo)-based title-only screening. Only a single study was falsely excluded by PICo-based title-only single screening in one of the 10 systematic reviews. The authors reported that “ventilation” was used in the title as an alternative term for oxygen therapy, and this was not listed in the MeSH (Medical Subject Headings) database nor found while searching other resources, and therefore, subject knowledge was needed to identify the study. They concluded that if authors use uncommon or ambiguous terminology in the abstract, this might lead to false exclusion [15].

#### Non-English publication language

One methods study with a case-study design, rated as low risk of bias, mentioned a publication language other than English as a characteristic for false exclusion during full-text screening. Busse et al. [16] evaluated a 10-question guide for English reviewers to assess the inclusion of non-English articles compared to native-language speakers. The authors reported false exclusion during the production of one systematic review of randomized controlled trials of fibromyalgia therapy including 53 publications published in 11 languages other than English. Six of the 53 full texts (11%) were falsely excluded by English-language reviewers due to being published in German (2/53), French, Turkish, Chinese, and Korean (1/53, respectively) languages [16].

## Discussion

To the best of our knowledge, this is the first systematic review that addresses methods to recover falsely excluded studies and study characteristics that can potentially predict a higher risk of false exclusions during literature screening. Our review identified three studies [10, 15–17]. One study reported on methods to recover studies falsely excluded during literature screening (KQ 1), all three included studies considered the characteristics of falsely excluded studies (KQ 2a), and no study was identified on predictors that could help identify studies at a high risk of being falsely excluded (KQ 2b). For KQ 1, only one methods study with a case study design that evaluated supplementary search methods was identified. Only reference list checking recovered 100% of the studies missed by dual literature screening; other supplementary search methods, such as contacting key authors, forward citation tracking, and hand searches of online journal websites, did not recover any falsely excluded studies. The study did not analyze the impact of recovered studies on conclusions or meta-analyses. [10, 17]. Three studies [10, 15–17] reported on the characteristics of falsely excluded studies. Two studies [10, 15, 17] reported false exclusions of up to 8% of studies due to uninformative titles and abstracts. Another study [16] reported false exclusion of 11% of studies due to the non-English publication language.

For rapid reviewers, the results underline the importance of reference list checking as well as considering uninformative titles/abstracts and non-English publications. It seems prudent that rapid review teams should check the reference lists of relevant publications to recover possible missed studies. Other supplementary search methods, such as contacting key authors, forward citation tracking, and hand searches of online journal websites, do not seem to be as effective. However, this evidence is derived from only a single study with a high risk of bias. Review team leaders should consider discussing uninformative titles and abstracts with screeners to avoid such false exclusions, perhaps by requesting that screeners include studies with uninformative titles or abstracts by default for a more in-depth evaluation at the full-text level. Journals and authors should strive to follow abstract reporting guidelines within the theme they are writing, considering the structure and content details of their abstract to ensure their studies are not falsely excluded. Journal editors and peer reviewers should also emphasize informative titles and abstracts. Additionally, the results show that authors should involve translation software or translators in the conduct of rapid and systematic reviews including non-English articles, either internally or through a research network. Generally, the growing use of automation software may prevent the false exclusion of studies [19]. This would require providing a clean, high-quality initial data set to train the algorithm, ensuring that any duplicates with conflicting decisions are removed and prespecifying records correctly as includes or excludes [19].

Overall, the quality and quantity of the included studies limit the evidence base of our systematic review. We identified three single-method studies on individual cases, assessing only 12 systematic reviews and 18 falsely excluded studies, that mostly address KQ 2. For KQ 1, we were only able to identify one method study with a case-study design. For KQ 2, we could only identify studies assessing the characteristics “Abstract content details” and “non-English publication language.” We were not able to identify studies addressing other characteristics or predictors of falsely excluded studies. We could not identify studies including predictive models for falsely excluded studies. It might be possible that reviewers falsely excluded studies due to other reasons (e.g., loss of concentration, reviewer’s experience) that were not formally addressed by this systematic review. Additionally, the review includes two high risks of bias studies of three included studies. We rated these two studies as having a high risk of bias mainly due to them analyzing a convenience case or case series and missing reports of the eligibility criteria and characteristics of the included cases. These flaws could have an influence on the reliability of our results. Therefore, the results should be cautiously taken into consideration.

Our systematic review has some methodological limitations. Although we applied a rigorous methodology according to Cochrane methods [11], we cannot rule out the possibility that we might have missed relevant studies. Another known threat to the validity of systematic reviews that we cannot exclude is publication bias. Although we searched for gray literature, relevant research on this topic might not be published due to nonsignificant results.

Based on our findings, there is an immense need for future research to evaluate supplementary search methods to recover studies falsely excluded during literature screening, particularly given the increased demand for rapid reviews employing literature screening shortcuts. No guidance document of any institution has addressed this issue. Studies assessing the overall impact of retrieving missed studies on conclusions and meta-analyses are also warranted. It is further unclear if falsely excluded studies are at risk of remaining permanently excluded. For example, a falsely excluded study may be retrieved again by reference list checking, however not screened a second time as it is discarded as duplicate. Additionally, further research on the characteristics and predictors of falsely excluded studies is needed. Thus, a methods study that analyzes a heterogeneous data set with a large quantity of screening decisions for possible predictors in a regression analysis or prediction model is warranted.

## Conclusions

We cannot draw any firm conclusion about the most reliable and most valid method to recover studies falsely excluded during literature screening, as the available evidence is limited to a single-case study. Furthermore, due to the limited evidence from two case studies and one case series, we can draw no firm conclusions on characteristics that might predict a higher risk of false exclusion.

## Supplementary Information

**Additional file 1. **PRISMA checklist.

**Additional file 2. **Search strategy for Medline, Science Citation Index Expanded, Social Sciences Citation Index, Current Contents Connect, Embase, Epistemonikos.org, and Information Science & Technology Abstracts.

**Additional file 3.** Search strategy for similar articles searches in PubMed, Google Scholar and Scopus.

**Additional file 4. **Excluded studies at the full-text level.

**Additional file 5. **Risk of bias of included studies.

## Data Availability

All data has been summarized and provided in the manuscript or supplementary files.

## Abbreviations

CADTH: Canadian Agency for Drugs and Technologies in Health
CI: Confidence interval
CONSORT: Consolidated Standards of Reporting Trials
DART: Digital Access to Research Theses
IMRaD: Introduction, Materials and Methods, Results, and Discussion and Conclusion
KQ: Key question
MeSH: Medical Subject Headings
*N*: Number
NA: Not applicable
NNR: Numbers needed to read
NR: Not reported
OR: Odds ratio
PRESS: Peer review of the electronic search strategy
PRISMA: Preferred Reporting Items for Systematic Reviews and Meta-Analyses
QUOROM: QUality Of Reporting Of Meta-analyses
RCT: Randomized controlled trial
SR: Systematic review
STARD: Standards for Reporting Diagnostic Accuracy
