# Supplementary material for: Characteristics and recovery methods of studies falsely excluded during literature screening—a systematic review
Source: Syst Rev. 2022 Nov 9;11:236. doi: 10.1186/s13643-022-02109-w (PMC9644550; doi:10.1186/s13643-022-02109-w)
Supplement: Supplementary file 2 — Additional file 2. Search strategy for Medline, Science Citation Index Expanded, Social Sciences Citation Index, Current Contents Connect, Embase, Epistemonikos.org, and Information Science & Technology Abstracts. [file 13643_2022_2109_MOESM2_ESM.docx]

**Additional file 2.** Search strategy of Medline, Science Citation Index Expanded, Social Sciences Citation Index, Current Contents Connect, Embase, Epistemonikos.org, and Information Science & Technology Abstracts

| Ovid MEDLINE(R) ALL 1946 to June 23, 2020 | |  |
| --- | --- | --- |
| **#** | **Searches** | **Results** |
| 1 | "review literature as topic"/ or systematic reviews as topic/ | 11091 |
| 2 | meta-analysis as topic/ | 17972 |
| 3 | ((systematic or rapid or evidence or accelerat* or scoping or knowledge) adj3 (review? or reviewing or synthes?s)).ti,kf. | 147207 |
| 4 | (meta analys?s or metaanalys?s or health technology assessment?).ti,kf. | 116824 |
| 5 | or/1-4 | 229629 |
| 6 | ((false* or incorrect* or inconsisten*) adj6 (irrelevant or exclu*) adj6 (study or studies or record? or article? or citation? or reference?)).ti,ab,kf. | 100 |
| 7 | (miss* adj2 (studies or study or record? or article? or citation? or reference?)).ti,ab,kf. | 2287 |
| 8 | ((false negativ* or overlook* or unidentif* or misidentif* or "not identified") adj4 (studies or study or record? or article? or citation? or reference?)).ti,ab,kf. | 3864 |
| 9 | or/6-8 | 6237 |
| 10 | 5 and 9 | 405 |
| 11 | ((citation? or abstract? or full text or fulltext or title? or article? or record?) adj3 screen*).ti,kf. | 117 |
| 12 | ((study or studies) adj2 identification).ti,kf. | 421 |
| 13 | (identif* adj3 (eligible or relevant) adj3 (studies or articles or citations or records or references)).ti,kf. | 15 |
| 14 | ((citation? or abstract? or full text or fulltext or title? or article? or record?) adj3 screen*).ab. /freq=2 | 1731 |
| 15 | ((study or studies) adj2 identification).ab. /freq=2 | 32 |
| 16 | (identif* adj3 (eligible or relevant) adj3 (studies or articles or citations or records or references)).ab. /freq=2 | 543 |
| 17 | ((supplement* or addition* or complement* or iterativ* or surveillance or simpl*) adj3 search*).ti,kf. | 211 |
| 18 | ((supplement* or addition* or complement* or iterativ* or surveillance or simpl*) adj3 search*).ab. /freq=2 | 666 |
| 19 | ((citation or manual or web*) adj search*).ti,kf. | 135 |
| 20 | ((citation or manual or web*) adj search*).ab. /freq=2 | 234 |
| 21 | (search engine? or Google).ti,kf. | 1863 |
| 22 | (search engine? or Google).ab. /freq=2 | 4021 |
| 23 | (trial regist* or study regist* or clinicaltrials* or CTgov or "CT.gov" or International Clinical Trials Registry Platform or ICTRP).ti,kf. | 895 |
| 24 | ((trial regist* or study regist* or clinicaltrials* or CTgov or "CT.gov" or International Clinical Trials Registry Platform or ICTRP) adj2 search*).ab. /freq=2 | 87 |
| 25 | ((related or similar) adj (articles or citations)).ti,kf. | 48 |
| 26 | ((related or similar) adj (articles or citations)).ab. /freq=2 | 90 |
| 27 | (hand search* or handsearch*).ti,kf. | 36 |
| 28 | (hand search* or handsearch*).ab. /freq=2 | 301 |
| 29 | ((citation or reference) adj1 (chas* or track* or list?)).ti,kf. | 71 |
| 30 | ((citation or reference) adj1 (chas* or track* or list?)).ab. /freq=2 | 367 |
| 31 | (contact* adj3 (author? or manufacturer? or companies or company or organi?ation? or stakeholder? or investigator? or trial coordinator?)).ti,kf. | 129 |
| 32 | (contact* adj3 (author? or manufacturer? or companies or company or organi?ation? or stakeholder? or investigator? or trial coordinator?)).ab. /freq=2 | 541 |
| 33 | (request* adj3 (information or studies or data or articles or evidence)).ti,kf. | 186 |
| 34 | (request* adj3 (information or studies or data or articles or evidence)).ab. /freq=2 | 430 |
| 35 | or/11-34 | 11871 |
| 36 | 5 and 35 | 3126 |
| 37 | methods.fs. | 3793649 |
| 38 | method*.ti,kf. | 544067 |
| 39 | method*.ab. /freq=2 | 1317661 |
| 40 | (task? or process* or approach* or tool? or software? or algorithm? or automat* or technology or technologies or technique? or technic? or machine learning or conduct* or develop* or undertak* or perform* or execut* or rule? or procedure? or guideline? or guidance?).ti,kf. | 2298048 |
| 41 | (approach* or technique? or technic? or performance).ab. /freq=2 | 1114156 |
| 42 | "Sensitivity and Specificity"/ | 346148 |
| 43 | (sensitivity or recall).ti,ab,kf. | 851559 |
| 44 | or/37-43 | 7527303 |
| 45 | 36 and 44 | 1698 |
| 46 | 10 or 45 | 2085 |
| 47 | limit 46 to yr="1999 -Current" | 2072 |

| Web of Science | |  |
| --- | --- | --- |
| 23 June 2029 | |  |
| Indexes=SCI-EXPANDED, SSCI Timespan=1999-2020 | | |
| Set | Result | Search |
| # 1 | 140621 | TI=("meta analys$s"  OR  metaanalys$s  OR  "health  technology  assessment$")  OR  AK=("meta analys$s"  OR  metaanalys$s  OR  "health  technology  assessment$") |
| # 2 | 161334 | TI=((systematic OR  rapid  OR  evidence  OR  accelerat*  OR  scoping  OR  knowledge)  NEAR/3  (review$ OR reviewing OR synthes$s) )  OR  AK=((systematic OR  rapid  OR  evidence  OR  accelerat*  OR  scoping  OR  knowledge)  NEAR/3  (review$ OR reviewing OR synthes$s) ) |
| # 3 | 246522 | #2  OR  #1 |
| # 4 | 137 | TS=((false* OR  incorrect*  OR  inconsisten*)  NEAR/6  (irrelevant OR exclu*)  NEAR/6  (study OR studies OR record$ OR article$ OR citation$ OR reference$) ) |
| # 5 | 3500 | TS=(miss* NEAR/1  (studies OR study OR record$ OR article$ OR citation$ OR reference$) ) |
| # 6 | 4987 | TS=(("false negativ*"  OR  overlook*  OR  unidentif*  OR  misidentif*  OR  "not  identified")  NEAR/3  (studies OR records OR articles OR citations OR references) ) |
| # 7 | 230 | TI=((citation$ OR  abstract$  OR  "full  text$"  OR  fulltext$  OR  title$  OR  article$  OR  record$)  NEAR/3  screen*)  OR  AK=((citation$ OR  abstract$  OR  "full  text$"  OR  fulltext$  OR  title$  OR  article$  OR  record$)  NEAR/3  screen*) |
| # 8 | 1 | TI=((study OR  studies)  NEAR/2  identification)  OR  AK=((study OR  studies)  NEAR/2  identification) |
| # 9 | 31 | TI=(identif* NEAR/3  (eligible OR relevant)  NEAR/3  (studies OR articles OR citations OR records OR references) )  OR  AK=(identif* NEAR/3  (eligible OR relevant)  NEAR/3  (studies OR articles OR citations OR records OR references) ) |
| # 10 | 617 | TI=((supplement* OR  addition*  OR  complement*  OR  iterativ*  OR  surveillance  OR  simpl*)  NEAR/3  search*)  OR  AK=((supplement* OR  addition*  OR  complement*  OR  iterativ*  OR  surveillance  OR  simpl*)  NEAR/3  search*) |
| # 11 | 1317 | TI=((citation OR  manual  OR  web*)  NEAR/1  search*)  OR  AK=((citation OR  manual  OR  web*)  NEAR/1  search*) |
| # 12 | 6381 | TI=("search engine$"  OR  google)  OR  AK=("search engine$"  OR  google) |
| # 13 | 949 | TI=("trial regist*"  OR  "study  regist*"  OR  clinicaltrials*  OR  ctgov  OR  "ct.gov"  OR  "international  clinical  trials  registry  platform"  OR  ictrp)  OR  AK=("trial regist*"  OR  "study  regist*"  OR  clinicaltrials*  OR  ctgov  OR  "ct.gov"  OR  "international  clinical  trials  registry  platform"  OR  ictrp) |
| # 14 | 115 | TI=((related OR  similar)  NEAR/1  (articles OR citations) )  OR  AK=((related OR  similar)  NEAR/1  (articles OR citations) ) |
| # 15 | 39 | TI=("hand search*"  OR  handsearch*)  OR  AK=("hand search*"  OR  handsearch*) |
| # 16 | 371 | TI=((citation OR  reference)  NEAR/1  (chas* OR track* OR list$) )  OR  AK=((citation OR  reference)  NEAR/1  (chas* OR track* OR list$) ) |
| # 17 | 531 | TI=(contact* NEAR/3  (author$ OR manufacturer$ OR companies OR company OR organi* OR stakeholder$ OR investigator$ OR "trial coordinator$") )  OR  AK=(contact* NEAR/3  (author$ OR manufacturer$ OR companies OR company OR organi* OR stakeholder$ OR investigator$ OR "trial coordinator$") ) |
| # 18 | 360 | TI=(request* NEAR/3  (information OR studies OR data OR articles OR evidence) )  OR  AK=(request* NEAR/3  (information OR studies OR data OR articles OR evidence) ) |
| # 19 | 19124 | #18  OR  #17  OR  #16  OR  #15  OR  #14  OR  #13  OR  #12  OR  #11  OR  #10  OR  #9  OR  #8  OR  #7  OR  #6  OR  #5  OR  #4 |
| # 20 | 575 | #19  AND  #3 |

| Current Contents Connect (Web of Science) | | |
| --- | --- | --- |
| 23 June 2029 | |  |
| Indexes=ABES, SBS, CM, LS, PCES, ECT, AH, BC, EC Timespan=1999-2020 | | |
| Set | Result | Search |
| # 1 | 83383 | TI=("meta analys$s"   OR   metaanalys$s   OR   "health   technology   assessment$")   OR   SU=("meta analys$s"   OR   metaanalys$s   OR   "health   technology   assessment$") |
| # 2 | 108058 | TI=((systematic OR   rapid   OR   evidence   OR   accelerat*   OR   scoping   OR   knowledge)   NEAR/3   (review$ OR reviewing OR synthes$s)  )   OR   SU=((systematic OR   rapid   OR   evidence   OR   accelerat*   OR   scoping   OR   knowledge)   NEAR/3   (review$ OR reviewing OR synthes$s)  ) |
| # 3 | 157170 | #2  OR  #1 |
| # 4 | 112 | TS=((false* OR   incorrect*   OR   inconsisten*)   NEAR/6   (irrelevant OR exclu*)    NEAR/6   (study OR studies OR record$ OR article$ OR citation$ OR reference$)  ) |
| # 5 | 3125 | TS=(miss* NEAR/1   (studies OR study OR record$ OR article$ OR citation$ OR reference$)  ) |
| # 6 | 4539 | TS=(("false negativ*"   OR   overlook*   OR   unidentif*   OR   misidentif*   OR   "not   identified")   NEAR/3   (studies OR records OR articles OR citations OR references)  ) |
| # 7 | 136 | TI=((citation$ OR   abstract$   OR   "full   text$"   OR   fulltext$   OR   title$   OR   article$   OR   record$)   NEAR/3   screen*)   OR   SU=((citation$ OR   abstract$   OR   "full   text$"   OR   fulltext$   OR   title$   OR   article$   OR   record$)   NEAR/3   screen*) |
| # 8 | 721 | TI=((study OR  studies)  NEAR/2  identification)  OR  SU=((study OR  studies)  NEAR/2  identification) |
| # 9 | 23 | TI=(identif* NEAR/3   (eligible OR relevant)    NEAR/3   (studies OR articles OR citations OR records OR references)  )   OR   SU=(identif* NEAR/3   (eligible OR relevant)    NEAR/3   (studies OR articles OR citations OR records OR references)  ) |
| # 10 | 423 | TI=((supplement* OR   addition*   OR   complement*   OR   iterativ*   OR   surveillance   OR   simpl*)   NEAR/3   search*)   OR   SU=((supplement* OR   addition*   OR   complement*   OR   iterativ*   OR   surveillance   OR   simpl*)   NEAR/3   search*) |
| # 11 | 822 | TI=((citation OR   manual   OR   web*)   NEAR/1   search*)   OR   SU=((citation OR   manual   OR   web*)   NEAR/1   search*) |
| # 12 | 3958 | TI=("search engine$"   OR   google)   OR   SU=("search engine$"   OR   google) |
| # 13 | 479 | TI=("trial regist*"   OR   "study   regist*"   OR   clinicaltrials*   OR   ctgov   OR   "ct.gov"   OR   "international   clinical   trials   registry   platform"   OR   ictrp)   OR   SU=("trial regist*"   OR   "study   regist*"   OR   clinicaltrials*   OR   ctgov   OR   "ct.gov"   OR   "international   clinical   trials   registry   platform"   OR   ictrp) |
| # 14 | 94 | TI=((related OR   similar)   NEAR/1   (articles OR citations)  )   OR   SU=((related OR   similar)   NEAR/1   (articles OR citations)  ) |
| # 15 | 25 | TI=("hand search*"   OR   handsearch*)   OR   SU=("hand search*"   OR   handsearch*) |
| # 16 | 177 | TI=((citation OR   reference)   NEAR/1   (chas* OR track* OR list$)  )   OR   SU=((citation OR   reference)   NEAR/1   (chas* OR track* OR list$)  ) |
| # 17 | 466 | TI=(contact* NEAR/3   (author$ OR manufacturer$ OR companies OR company OR organi* OR stakeholder$ OR investigator$ OR "trial coordinator$")  )   OR   SU=(contact* NEAR/3   (author$ OR manufacturer$ OR companies OR company OR organi* OR stakeholder$ OR investigator$ OR "trial coordinator$")  ) |
| # 18 | 279 | TI=(request* NEAR/3   (information OR studies OR data OR articles OR evidence)  )   OR   SU=(request* NEAR/3   (information OR studies OR data OR articles OR evidence)  ) |
| # 19 | 15153 | #18   OR   #17   OR   #16   OR   #15   OR   #14   OR   #13   OR   #12   OR   #11   OR   #10   OR   #9   OR   #8   OR   #7   OR   #6   OR   #5   OR   #4 |
| # 20 | 366 | #19  AND  #3 |

| Embase.com |  |  |
| --- | --- | --- |
| 26-Jun-20 |  |  |
| No. | Query | Results |
| #1 | 'systematic review (topic)'/exp/mj OR 'meta analysis (topic)'/exp/mj | 2401 |
| #2 | ((systematic OR rapid OR evidence OR accelerat* OR scoping OR knowledge) NEAR/3 (review$ OR reviewing OR synthes?s)):ti,kw | 174924 |
| #3 | 'meta analys?s':ti,kw OR metaanalys?s:ti,kw OR "health technology assessment$":ti,kw | 158633 |
| #4 | #1 OR #2 OR #3 | 263174 |
| #5 | ((false* OR incorrect* OR inconsisten*) NEAR/6 (irrelevant OR exclu*) NEAR/6 (study OR studies OR record$ OR article$ OR citation$ OR reference$)):ti,ab,kw | 181 |
| #6 | (miss* NEAR/2 (studies OR study OR record$ OR article$ OR citation$ OR reference$)):ti,ab,kw | 3327 |
| #7 | (('false negativ*' OR overlook* OR unidentif* OR misidentif* OR 'not identified') NEAR/4 (studies OR records OR articles OR citations OR references)):ti,ab,kw | 2555 |
| #8 | #5 OR #6 OR #7 | 9960 |
| #9 | #4 AND #8 | 563 |
| #10 | ((citation$ OR abstract$ OR "full text$" OR fulltext$ OR title$ OR article$ OR record$) NEAR/3 screen*):ti,kw | 163 |
| #11 | ((study OR studies) NEAR/2 identification):ti,kw | 558 |
| #12 | (identif* NEAR/3 (eligible OR relevant) NEAR/3 (studies OR articles OR citations OR records OR references)):ti,kw | 13 |
| #13 | ((supplement* OR addition* OR complement* OR iterativ* OR surveillance OR simpl*) NEAR/3 search*):ti,kw | 233 |
| #14 | ((citation OR manual OR web*) NEXT/1 search*):ti,kw | 165 |
| #15 | "search engine$":ti,kw OR google:ti,kw | 11635 |
| #16 | 'trial regist*':ti,kw OR 'study regist*':ti,kw OR clinicaltrials*:ti,kw OR ctgov:ti,kw OR 'ct.gov':ti,kw OR 'international clinical trials registry platform':ti,kw OR ictrp:ti,kw | 1249 |
| #17 | ((related OR similar) NEXT/1 (articles OR citations)):ti,kw | 65 |
| #18 | 'hand search*':ti,kw OR handsearch*:ti,kw | 42 |
| #19 | ((citation OR reference) NEAR/1 (chas* OR track* OR list$)):ti,kw | 67 |
| #20 | (contact* NEAR/3 (author$ OR manufacturer$ OR companies OR company OR organi?ation$ OR stakeholder$ OR investigator$ OR "trial coordinator$")):ti,kw | 85 |
| #21 | (request* NEAR/3 (information OR studies OR data OR articles OR evidence)):ti,kw | 235 |
| #22 | 'information retrieval'/mj | 9598 |
| #23 | 'documentation'/mj OR 'document examination'/mj | 9210 |
| #24 | #10 OR #11 OR #12 OR #13 OR #14 OR #15 OR #16 OR #17 OR #18 OR #19 OR #20 OR #21 OR #22 OR #23 | 23420 |
| #25 | #4 AND #24 | 407 |
| #26 | #9 OR #25 | 919 |
| #27 | #26 AND [1999-2020]/py | 910 |

| Epistemonikos.org | |  |
| --- | --- | --- |
| 23 June 2020 | |  |
| Set | Search | Result |
| 1 | title:((missing OR missed OR false* OR incorrect* OR inconsisten* OR "false negative" OR overlooked OR unidentif* OR misidentif* OR "not identified") AND (studies OR citations OR articles OR records OR references) AND (exclu* OR inclu* OR select*)) \| 1999-2020 | 2 |
| 2 | title:((citation* OR abstract* OR "full text" OR "full texts" OR fulltext* OR title* OR article* OR record*) AND (screen* OR identif*)) \| Broad synthesis, Systematic Review, Primary Study \| 1999-2020 | 82 |
| 3 | title:("study identification" OR (studies AND identif*)) \| 1999-2020 | 181 |
| 4 | title:((supplement* OR addition* OR complement* OR iterativ* OR surveillance OR simpl* OR citation OR manual OR web*) AND search*) \| 1999-2020 | 42 |
| 5 | title:("search engine" OR "search engines" OR google) \| 1999-2020 | 48 |
| 6 | title:(((trial OR study OR studies) AND regist*) OR clinicaltrials* OR ctgov OR "ct.gov" OR "international clinical trials registry platform" OR ictrp) \| Broad synthesis, Systematic Review \| 1999-2020 | 136 |
| 7 | title:("related articles" OR "related citations" OR "similar articles" OR "similar citations") \| 1999-2020 | 3 |
| 8 | title:("hand search" OR "hand searches" OR "hand searching" OR handsearch*) \| 1999-2020 | 13 |
| 9 | title:((citation OR reference) AND (chas* OR track* OR list*)) \| 1999-2020 | 2 |
| 10 | title:(contact* AND (author* OR manufacturer* OR companies OR company OR organisation* OR organization* OR stakeholder* OR investigator* OR coordinator*)) \| 1999-2020 | 3 |
| 11 | title:(request* AND (information OR studies OR data OR articles OR evidence)) \| 1999-2020 | 5 |
|  |  |  |
|  | Total (including duplicates) | 517 |

| Library, Information Science & Technology Abstracts | |  |  |
| --- | --- | --- | --- |
| 23 June 2020 | |  |  |
| # | Query | Limiters/Expanders | Results |
| S1 | meta analys#s OR metaanalys#s OR health technology assessment# | Search modes - Boolean/Phrase | 863 |
| S2 | ((systematic OR rapid OR evidence OR accelerat* OR scoping OR knowledge) N3 (review# OR reviewing OR synthes#s)) | Search modes - Boolean/Phrase | 3266 |
| S3 | S1 OR S2 | Search modes - Boolean/Phrase | 3505 |
| S4 | ((false* OR incorrect* OR inconsisten*) N5 (irrelevant OR exclu*) N5 (study OR studies OR record# OR article# OR citation# OR reference#)) | Search modes - Boolean/Phrase | 1 |
| S5 | (miss* N1 (studies OR study OR record# OR article# OR citation# OR reference#)) | Search modes - Boolean/Phrase | 213 |
| S6 | ((false negativ* OR overlook* OR unidentif* OR misidentif* OR not identified) N3 (studies OR records OR articles OR citations OR references)) | Search modes - Boolean/Phrase | 62 |
| S7 | TI ( ((citation# OR abstract# OR full text# OR fulltext# OR title# OR article# OR record#) N3 screen*) ) OR SU ( ((citation# OR abstract# OR full text# OR fulltext# OR title# OR article# OR record#) N3 screen*) ) | Search modes - Boolean/Phrase | 11 |
| S8 | TI ( ((study OR studies) N2 identification) ) OR SU ( ((study OR studies) N2 identification) ) | Search modes - Boolean/Phrase | 16 |
| S9 | TI ( (identif* N3 (eligible OR relevant) N3 (studies OR articles OR citations OR records OR references)) ) OR SU ( (identif* N3 (eligible OR relevant) N3 (studies OR articles OR citations OR records OR references)) ) | Search modes - Boolean/Phrase | 3 |
| S10 | DE "DATABASE selection" | Search modes - Boolean/Phrase | 34 |
| S11 | ((DE "CITATION networks") OR (DE "CITATION analysis" OR DE "CO-citation coupling")) OR (DE "CITATION indexes") | Search modes - Boolean/Phrase | 3780 |
| S12 | DE "SEARCH engines" OR DE "WEB search engines" | Search modes - Boolean/Phrase | 7295 |
| S13 | DE "BIBLIOGRAPHICAL citation searching" | Search modes - Boolean/Phrase | 62 |
| S14 | DE "FULL-text databases" | Search modes - Boolean/Phrase | 74 |
| S15 | DE "INFORMATION retrieval" OR DE "INFORMATION-seeking strategies" OR DE "INTERNET searching" | Search modes - Boolean/Phrase | 18930 |
| S16 | TI ( ((supplement* OR addition* OR complement* OR iterativ* OR surveillance OR simpl*) N3 search*) ) OR SU ( ((supplement* OR addition* OR complement* OR iterativ* OR surveillance OR simpl*) N3 search*) ) | Search modes - Boolean/Phrase | 105 |
| S17 | TI ( ((citation OR manual OR web*) W1 search*) ) OR SU ( ((citation OR manual OR web*) W1 search*) ) | Search modes - Boolean/Phrase | 3153 |
| S18 | TI ( search engine# OR google ) OR SU ( search engine# OR google ) | Search modes - Boolean/Phrase | 11880 |
| S19 | TI ( trial regist* OR study regist* OR clinicaltrials* OR ctgov OR "ct.gov" OR international clinical trials registry platform OR ictrp ) OR SU ( trial regist* OR study regist* OR clinicaltrials* OR ctgov OR ct.gov OR international clinical trials registry platform OR ictrp ) | Search modes - Boolean/Phrase | 134 |
| S20 | TI ( ((related OR similar) W0 (articles OR citations)) ) OR SU ( ((related OR similar) W0 (articles OR citations)) ) | Search modes - Boolean/Phrase | 15 |
| S21 | TI ( hand search* OR handsearch* ) OR SU ( hand search* OR handsearch* ) | Search modes - Boolean/Phrase | 16 |
| S22 | TI ( ((citation OR reference) N1 (chas* OR track* OR list#)) ) OR SU ( ((citation OR reference) N1 (chas* OR track* OR list#)) ) | Search modes - Boolean/Phrase | 61 |
| S23 | TI ( (contact* N3 (author# OR manufacturer# OR companies OR company OR organi?ation# OR stakeholder# OR investigator# OR trial coordinator#)) ) OR SU ( (contact* N3 (author# OR manufacturer# OR companies OR company OR organi?ation# OR stakeholder# OR investigator# OR trial coordinator#)) ) | Search modes - Boolean/Phrase | 3 |
| S24 | TI ( (request* N3 (information OR studies OR data OR articles OR evidence)) ) OR SU ( (request* N3 (information OR studies OR data OR articles OR evidence)) ) | Search modes - Boolean/Phrase | 100 |
| S25 | S4 OR S5 OR S6 OR S7 OR S8 OR S9 OR S10 OR S11 OR S12 OR S13 OR S14 OR S15 OR S16 OR S17 OR S18 OR S19 OR S20 OR S21 OR S22 OR S23 OR S24 | Search modes - Boolean/Phrase | 32041 |
| S26 | S3 AND S25 | Search modes - Boolean/Phrase | 269 |
| S27 | S3 AND S25 | Limiters - Publication Date: 19990101-20201231 | 264 |
