# Supplementary material for: Characteristics and recovery methods of studies falsely excluded during literature screening—a systematic review
Source: Syst Rev. 2022 Nov 9;11:236. doi: 10.1186/s13643-022-02109-w (PMC9644550; doi:10.1186/s13643-022-02109-w)
Supplement: Supplementary file 3 — Additional file 3. Search strategy for similar articles searches in PubMed, Google Scholar and Scopus. [file 13643_2022_2109_MOESM3_ESM.docx]

**Additional file 2.** Search strategy of similar articles searches in Pubmed, Google Scholar and Scopus

| Similar Articles (First 20 linked references for every seed article) |  |  |
| --- | --- | --- |
| Pubmed |  |  |
| 28 Jan 2021 |  |  |
|  |  |  |
| References | PMIDs | Similar Articles |
| Affengruber, L., G. Wagner, S. Waffenschmidt, S. K. Lhachimi, B. Nussbaumer-Streit, K. Thaler, U. Griebler, I. Klerings and G. Gartlehner (2020). "Combining abbreviated literature searches with single-reviewer screening: three case studies of rapid reviews." Systematic Reviews 9(1): 162. | 32682442 | 89 |
| Doust, J. A., E. Pietrzak, S. Sanders and P. P. Glasziou (2005). "Identifying studies for systematic reviews of diagnostic tests was difficult due to the poor sensitivity and precision of methodologic filters and the lack of information in the abstract." Journal of Clinical Epidemiology 58(5): 444-449. | 15845330 | 54 |
| Edwards, P., M. Clarke, C. DiGuiseppi, S. Pratap, I. Roberts and R. Wentz (2002). "Identification of randomized controlled trials in systematic reviews: Accuracy and reliability of screening records." Statistics in Medicine 21(11): 1635-1640. | 12111924 | 174 |
| Feehan, L. M., C. A. Beck, S. R. Harris, D. L. MacIntyre and L. C. Li (2011). "Exercise prescription after fragility fracture in older adults: a scoping review." Osteoporosis International 22(5): 1289-1322. | 20967425 | 48 |
| Gartlehner, G., L. Affengruber, V. Titscher, A. Noel-Storr, G. Dooley, N. Ballarini and F. Konig (2020). "Single-reviewer abstract screening missed 13 percent of relevant studies: a crowd-based, randomized controlled trial." Journal of Clinical Epidemiology 121: 20-28. | 31972274 | 103 |
| Gates, A., M. Gates, M. Sebastianski, S. Guitard, S. A. Elliott and L. Hartling (2020). "The semi-automation of title and abstract screening: a retrospective exploration of ways to leverage Abstrackr's relevance predictions in systematic and rapid reviews." BMC Medical Research Methodology 20(1): 139. | 32493228 | 95 |
| Janssens, A. C. and M. Gwinn (2015). "Novel citation-based search method for scientific literature: application to meta-analyses." BMC Medical Research Methodology 15: 84. | 26462491 | 54 |
| Mortensen, M. L., G. P. Adam, T. A. Trikalinos, T. Kraska and B. C. Wallace (2017). "An exploration of crowdsourcing citation screening for systematic reviews." Research Synthesis Methods 8(3): 366-386. | 28677322 | 100 |
| Ng, L., V. Pitt, K. Huckvale, O. Clavisi, T. Turner, R. Gruen and J. H. Elliott (2014). "Title and Abstract Screening and Evaluation in Systematic Reviews (TASER): a pilot randomised controlled trial of title and abstract screening by medical students." Systematic Reviews 3: 121. | 25335439 | 46 |
| Rosen, L. and R. Suhami (2016). "The art and science of study identification: a comparative analysis of two systematic reviews." BMC Medical Research Methodology 16: 24. | 26911333 | 99 |
| Stoll, C. R. T., S. Izadi, S. Fowler, P. Green, J. Suls and G. A. Colditz (2019). "The value of a second reviewer for study selection in systematic reviews." Research Synthesis Methods 10(4): 539-545. | 31272125 | 113 |
| Waffenschmidt, S., M. Knelangen, W. Sieben, S. Buhn and D. Pieper (2019). "Single screening versus conventional double screening for study selection in systematic reviews: a methodological systematic review." BMC Medical Research Methodology 19(1): 132. | 31253092 | 122 |
| Wang, Z., T. Nayfeh, J. Tetzlaff, P. O'Blenis and M. H. Murad (2020). "Error rates of human reviewers during abstract screening in systematic reviews." PLoS ONE [Electronic Resource] 15(1): e0227742. | 31935267 | 162 |
|  |  |  |
| Total: First 20 linked references for every seed article (without duplicates) |  | 180 |

| Similar Articles (First 20 linked references for every seed article) |  |
| --- | --- |
| Google Scholar |  |
| 28 Jan 2021 |  |
|  |  |
| References |  |
| Affengruber, L., G. Wagner, S. Waffenschmidt, S. K. Lhachimi, B. Nussbaumer-Streit, K. Thaler, U. Griebler, I. Klerings and G. Gartlehner (2020). "Combining abbreviated literature searches with single-reviewer screening: three case studies of rapid reviews." Systematic Reviews 9(1): 162. |  |
| Doust, J. A., E. Pietrzak, S. Sanders and P. P. Glasziou (2005). "Identifying studies for systematic reviews of diagnostic tests was difficult due to the poor sensitivity and precision of methodologic filters and the lack of information in the abstract." Journal of Clinical Epidemiology 58(5): 444-449. |  |
| Edwards, P., M. Clarke, C. DiGuiseppi, S. Pratap, I. Roberts and R. Wentz (2002). "Identification of randomized controlled trials in systematic reviews: Accuracy and reliability of screening records." Statistics in Medicine 21(11): 1635-1640. |  |
| Feehan, L. M., C. A. Beck, S. R. Harris, D. L. MacIntyre and L. C. Li (2011). "Exercise prescription after fragility fracture in older adults: a scoping review." Osteoporosis International 22(5): 1289-1322. |  |
| Gartlehner, G., L. Affengruber, V. Titscher, A. Noel-Storr, G. Dooley, N. Ballarini and F. Konig (2020). "Single-reviewer abstract screening missed 13 percent of relevant studies: a crowd-based, randomized controlled trial." Journal of Clinical Epidemiology 121: 20-28. |  |
| Gates, A., M. Gates, M. Sebastianski, S. Guitard, S. A. Elliott and L. Hartling (2020). "The semi-automation of title and abstract screening: a retrospective exploration of ways to leverage Abstrackr's relevance predictions in systematic and rapid reviews." BMC Medical Research Methodology 20(1): 139. |  |
| Janssens, A. C. and M. Gwinn (2015). "Novel citation-based search method for scientific literature: application to meta-analyses." BMC Medical Research Methodology 15: 84. |  |
| Mortensen, M. L., G. P. Adam, T. A. Trikalinos, T. Kraska and B. C. Wallace (2017). "An exploration of crowdsourcing citation screening for systematic reviews." Research Synthesis Methods 8(3): 366-386. |  |
| Ng, L., V. Pitt, K. Huckvale, O. Clavisi, T. Turner, R. Gruen and J. H. Elliott (2014). "Title and Abstract Screening and Evaluation in Systematic Reviews (TASER): a pilot randomised controlled trial of title and abstract screening by medical students." Systematic Reviews 3: 121. |  |
| Rosen, L. and R. Suhami (2016). "The art and science of study identification: a comparative analysis of two systematic reviews." BMC Medical Research Methodology 16: 24. |  |
| Stoll, C. R. T., S. Izadi, S. Fowler, P. Green, J. Suls and G. A. Colditz (2019). "The value of a second reviewer for study selection in systematic reviews." Research Synthesis Methods 10(4): 539-545. |  |
| Waffenschmidt, S., M. Knelangen, W. Sieben, S. Buhn and D. Pieper (2019). "Single screening versus conventional double screening for study selection in systematic reviews: a methodological systematic review." BMC Medical Research Methodology 19(1): 132. |  |
| Wang, Z., T. Nayfeh, J. Tetzlaff, P. O'Blenis and M. H. Murad (2020). "Error rates of human reviewers during abstract screening in systematic reviews." PLoS ONE [Electronic Resource] 15(1): e0227742. |  |
|  |  |
| Total: First 20 linked references for every seed article (without duplicates) | 230 |

| Forward citation tracking |  |
| --- | --- |
| Scopus |  |
| 28 Jan 2021 |  |
|  |  |
| References | Cited by |
| Affengruber, L., G. Wagner, S. Waffenschmidt, S. K. Lhachimi, B. Nussbaumer-Streit, K. Thaler, U. Griebler, I. Klerings and G. Gartlehner (2020). "Combining abbreviated literature searches with single-reviewer screening: three case studies of rapid reviews." Systematic Reviews 9(1): 162. | 1 |
| Doust, J. A., E. Pietrzak, S. Sanders and P. P. Glasziou (2005). "Identifying studies for systematic reviews of diagnostic tests was difficult due to the poor sensitivity and precision of methodologic filters and the lack of information in the abstract." Journal of Clinical Epidemiology 58(5): 444-449. | 67 |
| Edwards, P., M. Clarke, C. DiGuiseppi, S. Pratap, I. Roberts and R. Wentz (2002). "Identification of randomized controlled trials in systematic reviews: Accuracy and reliability of screening records." Statistics in Medicine 21(11): 1635-1640. | 164 |
| Feehan, L. M., C. A. Beck, S. R. Harris, D. L. MacIntyre and L. C. Li (2011). "Exercise prescription after fragility fracture in older adults: a scoping review." Osteoporosis International 22(5): 1289-1322. | 10 |
| Gartlehner, G., L. Affengruber, V. Titscher, A. Noel-Storr, G. Dooley, N. Ballarini and F. Konig (2020). "Single-reviewer abstract screening missed 13 percent of relevant studies: a crowd-based, randomized controlled trial." Journal of Clinical Epidemiology 121: 20-28. | 15 |
| Gates, A., M. Gates, M. Sebastianski, S. Guitard, S. A. Elliott and L. Hartling (2020). "The semi-automation of title and abstract screening: a retrospective exploration of ways to leverage Abstrackr's relevance predictions in systematic and rapid reviews." BMC Medical Research Methodology 20(1): 139. | 2 |
| Janssens, A. C. and M. Gwinn (2015). "Novel citation-based search method for scientific literature: application to meta-analyses." BMC Medical Research Methodology 15: 84. | 19 |
| Mortensen, M. L., G. P. Adam, T. A. Trikalinos, T. Kraska and B. C. Wallace (2017). "An exploration of crowdsourcing citation screening for systematic reviews." Research Synthesis Methods 8(3): 366-386. | 21 |
| Ng, L., V. Pitt, K. Huckvale, O. Clavisi, T. Turner, R. Gruen and J. H. Elliott (2014). "Title and Abstract Screening and Evaluation in Systematic Reviews (TASER): a pilot randomised controlled trial of title and abstract screening by medical students." Systematic Reviews 3: 121. | 12 |
| Rosen, L. and R. Suhami (2016). "The art and science of study identification: a comparative analysis of two systematic reviews." BMC Medical Research Methodology 16: 24. | 7 |
| Stoll, C. R. T., S. Izadi, S. Fowler, P. Green, J. Suls and G. A. Colditz (2019). "The value of a second reviewer for study selection in systematic reviews." Research Synthesis Methods 10(4): 539-545. | 11 |
| Waffenschmidt, S., M. Knelangen, W. Sieben, S. Buhn and D. Pieper (2019). "Single screening versus conventional double screening for study selection in systematic reviews: a methodological systematic review." BMC Medical Research Methodology 19(1): 132. | 26 |
| Wang, Z., T. Nayfeh, J. Tetzlaff, P. O'Blenis and M. H. Murad (2020). "Error rates of human reviewers during abstract screening in systematic reviews." PLoS ONE [Electronic Resource] 15(1): e0227742. | 3 |
|  |  |
| Total (without duplicates) | 329 |
