# Supplementary material for: Characteristics and recovery methods of studies falsely excluded during literature screening—a systematic review
Source: Syst Rev. 2022 Nov 9;11:236. doi: 10.1186/s13643-022-02109-w (PMC9644550; doi:10.1186/s13643-022-02109-w)
Supplement: Supplementary file 4 — Additional file 4. Excluded studies at the full-text level. [file 13643_2022_2109_MOESM4_ESM.docx]

**Additional file 4.** Excluded studies at the full-text level

**Wrong topic**

1. Aabenhus R, Jensen JU, Cals JW. Incorrect inclusion of individual studies and methodological flaws in systematic review and meta-analysis. The British journal of general practice : the journal of the Royal College of General Practitioners. 2014;64(622):221-2.

2. Al Hajeri A, Al Sayyad J, Eisinga A. Handsearching the EMHJ for reports of randomized controlled trials by UK Cochrane Centre [Bahrain]. 2006.

3. Al-Hajeri AA, Fedorowicz Z, Amin FA, Eisinga A. The handsearching of 2 medical journals of Bahrain for reports of randomized controlled trials. Saudi Med J. 2006;27(4):526-30.

4. Arevalo-Rodriguez I, Moreno-Nunez P, Nussbaumer-Streit B, Steingart KR, Peña LdMG, Buitrago-Garcia D, et al. Rapid reviews of medical tests used many similar methods to systematic reviews but key items were rarely reported: a scoping review. Journal of clinical epidemiology. 2019;116:98-105.

5. Armstrong R, Jackson N, Doyle J, Waters E, Howes F. It's in your hands: the value of handsearching in conducting systematic reviews of public health interventions. J Public Health (Oxf). 2005;27(4):388-91.

6. Bagheri E, Rios P, Pourmasoumi A, Robson RC, Hwee J, Isaranuwatchai W, et al. Improving the conduct of systematic reviews: a process mining perspective. Journal of clinical epidemiology. 2018;103:101-11.

7. Barnish M. DOES A RAPID REVIEW VERSION OF A LARGE EPIDEMIOLOGICAL SYSTEMATIC REVIEW FAIL TO IDENTIFY MANY ELIGIBLE STUDIES, AND WHAT IMPLICATIONS DOES THIS HAVE FOR THE RESULTS OF THE REVIEW? J Epidemiol Community Health. 2019;73:A40-A1.

8. Baudard M, Yavchitz A, Ravaud P, Perrodeau E, Boutron I. Impact of searching clinical trial registries in systematic reviews of pharmaceutical treatments: methodological systematic review and reanalysis of meta-analyses. Bmj. 2017;356:j448.

9. Beckmann M, von Wehrden H. Where you search is what you get: literature mining - Google Scholar versus Web of Science using a data set from a literature search in vegetation science. J Veg Sci. 2012;23(6):1197-9.

10. Bekhuis T, Demner-Fushman D. Screening nonrandomized studies for medical systematic reviews: a comparative study of classifiers. Artif Intell Med. 2012;55(3):197-207.

11. Belter C. A relevance ranking method for citation-based search results. Scientometrics. 2017;112(2):731-46.

12. Belter CW. Citation analysis as a literature search method for systematic reviews. Journal of the Association for Information Science & Technology. 2016;67(11):2766-77.

13. Bereczki D, Gesztelyi G. A Hungarian example for handsearching specialized national healthcare journals of small countries for controlled trials. Is it worth the trouble? Health libraries review. 2000;17(3):144-7.

14. Blackhall K. Finding studies for inclusion in systematic reviews of interventions for injury prevention the importance of grey and unpublished literature. Injury prevention : journal of the International Society for Child and Adolescent Injury Prevention. 2007;13(5):359.

15. Blanc X, Collet T-H, Auer R, Iriarte P, Krause J, Légaré F, et al. Retrieval of Publications Addressing Shared Decision Making: An Evaluation of Full-Text Searches on Medical Journal Websites. Journal of Medical Internet Research. 2015;17(4):1-.

16. Blümle A, Antes G. [Handsearching for randomized controlled clinical trials in German medical journals]. Deutsche medizinische Wochenschrift (1946). 2008;133(6):230-4.

17. Blümle A, Antes G, Diener MK. [Hand searching for controlled clinical trials in German surgical journals. A contribution to evidence-based surgery]. Der Chirurg; Zeitschrift für alle Gebiete der operativen Medizen. 2007;78(11):1052-7.

18. Boeker M, Vach W, Motschall E. Google Scholar as replacement for systematic literature searches: good relative recall and precision are not enough. BMC Med Res Methodol. 2013;13:131.

19. Bramer WM, Giustini D, Kramer BM. Comparing the coverage, recall, and precision of searches for 120 systematic reviews in Embase, MEDLINE, and Google Scholar: a prospective study. Syst. 2016;5:39.

20. Briscoe S, Bethel A, Rogers M. Conduct and reporting of citation searching in Cochrane systematic reviews: A cross-sectional study. Res. 2020;11(2):169-80.

21. Brueton V, Tierney JF, Stenning S, Rait G. Identifying additional studies for a systematic review of retention strategies in randomised controlled trials: making contact with trials units and trial methodologists. Systematic reviews. 2017;6(1):167.

22. Choong MK, Tsafnat G. Role of citation tracking in updating of systematic reviews. AMIA Summits Transl Sci Proc. 2014;2014:18.

23. Cleo G, Scott AM, Islam F, Julien B, Beller E. Usability and acceptability of four systematic review automation software packages: a mixed method design. Syst. 2019;8(1):145.

24. Combs T, Atakpo P, Vassar M. A review of clinical trial registry use in dermatology systematic reviews. British Journal of Dermatology. 2018;178(5):1218-9.

25. Cooper C, Booth A, Varley-Campbell J, Britten N, Garside R. Defining the process to literature searching in systematic reviews: a literature review of guidance and supporting studies. BMC Med Res Methodol. 2018;18(1):85.

26. Cooper C, Lovell R, Husk K, Booth A, Garside R. Supplementary search methods were more effective and offered better value than bibliographic database searching: A case study from public health and environmental enhancement. Res. 2018;9(2):195-223.

27. Cormack GV, Grossman MR, editors. Technology-assisted review in empirical medicine: Waterloo participation in CLEF eHealth 20172017: CEUR-WS.

28. Craane B, Dijkstra PU, Stappaerts K, De Laat A. Methodological quality of a systematic review on physical therapy for temporomandibular disorders: influence of hand search and quality scales. Clin Oral Investig. 2012;16(1):295-303.

29. Crumley ET, Wiebe N, Cramer K, Klassen TP, Hartling L. Which resources should be used to identify RCT/CCTs for systematic reviews: a systematic review. BMC Med Res Methodol. 2005;5:24.

30. Demner-Fushman D, Hauser S, Thoma G. The role of title, metadata and abstract in identifying clinically relevant journal articles. AMIA Annual Symposium proceedings / AMIA Symposium AMIA Symposium. 2005:191-5.

31. Duffy S, de Kock S, Misso K, Noake C, Ross J, Stirk L. Supplementary searches of PubMed to improve currency of MEDLINE and MEDLINE In-Process searches via Ovid. J Med Libr Assoc. 2016;104(4):309-12.

32. Dunn AG, Coiera E, Bourgeois FT. Unreported links between trial registrations and published articles were identified using document similarity measures in a cross-sectional analysis of ClinicalTrials.gov. J Clin Epidemiol. 2018;95:94-101.

33. Dykeman J, Faris P, Dykeman M, Jette N, Wiebe S. Evaluating the use of capture-mark-recapture for determining the number of missing articles in systematic reviews. American Journal of Epidemiology. 2012;175:S20.

34. Ewald H, Klerings I, Wagner G, Heise TL, Dobrescu AI, Armijo-Olivo S, et al. Abbreviated and comprehensive literature searches led to identical or very similar effect estimates: meta-epidemiological study. Journal of Clinical Epidemiology. 2020.

35. Fergusson D, Laupacis A, Rachid Salmi L, McAlister FA, Huet C. What should be included in meta-analyses? An exploration methodological issues using the ISPOT meta-analyses. International Journal of Technology Assessment in Health Care. 2000;16(4):1109-19.

36. Freeman MK, Lauderdale SA, Kendrach MG, Woolley TW. Google Scholar versus PubMed in locating primary literature to answer drug-related questions. Annals of Pharmacotherapy. 2009;43(3):478-84.

37. Gates A, Guitard S, Pillay J, Elliott SA, Dyson MP, Newton AS, et al. Performance and usability of machine learning for screening in systematic reviews: a comparative evaluation of three tools. Syst. 2019;8(1):278.

38. Giummarra MJ, Lau G, Gabbe BJ. Evaluation of text mining to reduce screening workload for injury-focused systematic reviews. Inj Prev. 2020;26(1):55-60.

39. Glanville JM, Duffy S, McCool R, Varley D. Searching ClinicalTrials.gov and the International Clinical Trials Registry Platform to inform systematic reviews: what are the optimal search approaches? J Med Libr Assoc. 2014;102(3):177-83.

40. Godin K, Stapleton J, Kirkpatrick SI, Hanning RM, Leatherdale ST. Applying systematic review search methods to the grey literature: a case study examining guidelines for school-based breakfast programs in Canada. Syst. 2015;4:138.

41. Golder S, Loke YK, Zorzela L. Comparison of search strategies in systematic reviews of adverse effects to other systematic reviews. Health Information & Libraries Journal. 2014;31(2):92-105.

42. Golder S, McIntosh HM, Loke Y. Identifying systematic reviews of the adverse effects of health care interventions. BMC Med Res Methodol. 2006;6:22.

43. Gusenbauer M, Haddaway NR. Which academic search systems are suitable for systematic reviews or meta-analyses? Evaluating retrieval qualities of Google Scholar, PubMed, and 26 other resources. Res. 2020;11(2):181-217.

44. Hamel C, Kelly SE, Thavorn K, Rice DB, Wells GA, Hutton B. An evaluation of DistillerSR's machine learning-based prioritization tool for title/abstract screening - impact on reviewer-relevant outcomes. BMC Med Res Methodol. 2020;20(1):256.

45. Helmer D, Savoie I, Green C, Kazanjian A. Evidence-based practice: extending the search to find material for the systematic review. Bull Med Libr Assoc. 2001;89(4):346-52.

46. Hempel S, Shetty KD, Shekelle PG, Rubenstein LV, Danz MS, Johnsen B, et al. AHRQ Methods for Effective Health Care. Rockville (MD): Agency for Healthcare Research and Quality (US); 2012.

47. Herman SW. Google Scholar Could Be Used as a Stand-Alone Resource for Systematic Reviews. Evidence Based Library & Information Practice. 2015;10(2):147-9.

48. Howard BE, Phillips J, Tandon A, Maharana A, Elmore R, Mav D, et al. SWIFT-Active Screener: Accelerated document screening through active learning and integrated recall estimation. Environ Int. 2020;138:105623.

49. Janssens A, Gwinn M, Brockman JE, Powell K, Goodman M. Novel citation-based search method for scientific literature: a validation study. BMC Med Res Methodol. 2020;20(1):25.

50. Ji X, Yen PY. Using MEDLINE Elemental Similarity to Assist in the Article Screening Process for Systematic Reviews. JMIR Med Inform. 2015;3(3):e28.

51. Kelley GA, Kelley KS, Tran ZV. Retrieval of missing data for meta-analysis: A practical example. International Journal of Technology Assessment in Health Care. 2004;20(3):296-9.

52. Khabsa M, Elmagarmid A, Ilyas I, Hammady H, Ouzzani M. Learning to identify relevant studies for systematic reviews using random forest and external information. Mach Learn. 2016;102(3):465-82.

53. Levay P, Ainsworth N, Kettle R, Morgan A. Identifying evidence for public health guidance: a comparison of citation searching with Web of Science and Google Scholar. Res. 2016;7(1):34-45.

54. Linder SK, Kamath GR, Pratt GF, Saraykar SS, Volk RJ. Citation searches are more sensitive than keyword searches to identify studies using specific measurement instruments. J Clin Epidemiol. 2015;68(4):412-7.

55. Lopes R, Gauthier G, Akhtar O, Atanasov P. PERFORMANCE OF AUTOMATED SCREENING OF CITATIONS COMPARED TO HUMAN REVIEWERS IN SYSTEMATIC LITERATURE REVIEWS: A SYSTEMATIC LITERATURE REVIEW. Value in Health. 2018;21:S367.

56. Mahtani KR, Heneghan C, Aronson J. Single screening or double screening for study selection in systematic reviews? BMJ Evid Based Med. 2020;25(4):149-50.

57. Mateen FJ, Oh J, Tergas AI, Bhayani NH, Kamdar BB. Titles versus titles and abstracts for initial screening of articles for systematic reviews. Clin Epidemiol. 2013;5:89-95.

58. Matthews EJ, Edwards AG, Barker J, Bloor M, Covey J, Hood K, et al. Efficient literature searching in diffuse topics: lessons from a systematic review of research on communicating risk to patients in primary care. Health libraries review. 1999;16(2):112-20.

59. Mattivi JT, Buchberger B. [Rapid Reviews: Sisyphos' Salvation? - An Inventory]. Gesundheitswesen. 2017;79(5):438-42.

60. McDonald S, Lefebvre C, Antes G, Galandi D, Gøtzsche P, Hammarquist C, et al. The contribution of handsearching European general health care journals to the Cochrane Controlled Trials Register. Evaluation & the health professions. 2002;25(1):65-75.

61. Moreno SG, Sutton AJ, Turner EH, Abrams KR, Cooper NJ, Palmer TM, et al. Novel methods to deal with publication biases: secondary analysis of antidepressant trials in the FDA trial registry database and related journal publications. BMJ (Clinical research ed). 2009;339(7719):b2981.

62. Nama N, Barrowman N, O'Hearn K, Sampson M, Zemek R, McNally JD. Quality control for crowdsourcing citation screening: the importance of assessment number and qualification set size. J Clin Epidemiol. 2020;122:160-2.

63. Nama N, Iliriani K, Xia MY, Chen BP, Zhou LL, Pojsupap S, et al. A pilot validation study of crowdsourcing systematic reviews: update of a searchable database of pediatric clinical trials of high-dose vitamin D. Transl. 2017;6(1):18-26.

64. Nama N, Sampson M, Barrowman N, O'Hearn K, Sandarage R, Menon K, et al. Validation of crowdsourcing for citation screening in systematic reviews. BMJ Evidence-Based Medicine. 2019;24:A26.

65. Noel-Storr A, Dooley G, Affengruber L, Gartlehner G. Citation screening using crowdsourcing and machine learning produced accurate results: Evaluation of Cochrane's modified Screen4Me service. J Clin Epidemiol. 2021;130:23-31.

66. Norgaard MF, Grauslund J. Automated Screening for Diabetic Retinopathy - A Systematic Review. Ophthalmic Res. 2018;60(1):9-17.

67. Norman CR, Gargon E, Leeflang MMG, Neveol A, Williamson PR. Evaluation of an automatic article selection method for timelier updates of the Comet Core Outcome Set database. Database (Oxford). 2019;01:01.

68. Pardo-Hernandez H, Urrutia G, Barajas-Nava LA, Buitrago-Garcia D, Garzon JV, Martinez-Zapata MJ, et al. BADERI: an online database to coordinate handsearching activities of controlled clinical trials for their potential inclusion in systematic reviews. Trials. 2017;18(1):273.

69. Piasecki J, Waligora M, Dranseika V. Google Search as an Additional Source in Systematic Reviews. Science and engineering ethics. 2018;24(2):809-10.

70. Plevier JWM, Schoones JW. Optimization of literature searching. Journal of Clinical Epidemiology. 2012;65(9):1027-8.

71. Polanin JR, Pigott TD, Espelage DL, Grotpeter JK. Best practice guidelines for abstract screening large-evidence systematic reviews and meta-analyses. Res Synth Methods. 2019;10(3):330-42.

72. Preston L, Carroll C, Gardois P, Paisley S, Kaltenthaler E. Improving search efficiency for systematic reviews of diagnostic test accuracy: an exploratory study to assess the viability of limiting to MEDLINE, EMBASE and reference checking. Syst. 2015;4:82.

73. Robson RC, Pham B, Hwee J, Thomas SM, Rios P, Page MJ, et al. Few studies exist examining methods for selecting studies, abstracting data, and appraising quality in a systematic review. J Clin Epidemiol. 2019;106:121-35.

74. Royle P, Waugh N. A simplified search strategy for identifying randomised controlled trials for systematic reviews of health care interventions: a comparison with more exhaustive strategies. BMC Med Res Methodol. 2005;5:23.

75. Sampson M, Barrowman NJ, Moher D, Clifford TJ, Platt RW, Morrison A, et al. Can electronic search engines optimize screening of search results in systematic reviews: an empirical study. BMC Med Res Methodol. 2006;6:7.

76. Sampson M, de Bruijn B, Urquhart C, Shojania K. Complementary approaches to searching MEDLINE may be sufficient for updating systematic reviews. J Clin Epidemiol. 2016;78:108-15.

77. Sampson M, Zhang L, Morrison A, Barrowman NJ, Clifford TJ, Platt RW, et al. An alternative to the hand searching gold standard: validating methodological search filters using relative recall. BMC Med Res Methodol. 2006;6:33.

78. Savoie I, Helmer D, Green CJ, Kazanjian A. Beyond Medline: reducing bias through extended systematic review search. Int J Technol Assess Health Care. 2003;19(1):168-78.

79. Si JH, Li Y, Shang HC. How to conduct literature retrieval of systematic review in proquest dialog. Chinese Journal of Evidence-Based Medicine. 2015;15(5):612-6.

80. Stansfield C, Kavanagh J, Rees R, Gomersall A, Thomas J. The selection of search sources influences the findings of a systematic review of people's views: a case study in public health. BMC Med Res Methodol. 2012;12:55.

81. Tsafnat G, Glasziou P, Karystianis G, Coiera E. Automated screening of research studies for systematic reviews using study characteristics. Syst. 2018;7(1):64.

82. Valderas JM, Kotzeva A, Espallargues M, Guyatt G, Ferrans CE, Halyard MY, et al. A relevant study was missed in our systematic review on the impact of patient-reported outcomes in clinical practice. Quality of Life Research. 2008;17(6):965-6.

83. van Enst WA, Scholten RJ, Hooft L. Identification of additional trials in prospective trial registers for Cochrane systematic reviews. PLoS ONE. 2012;7(8):e42812.

84. Vassar M, Atakpo P, Kash MJ. Manual search approaches used by systematic reviewers in dermatology. J Med Libr Assoc. 2016;104(4):302-4.

85. Waffenschmidt S, Janzen T, Hausner E, Kaiser T. Simple search techniques in PubMed are potentially suitable for evaluating the completeness of systematic reviews. J Clin Epidemiol. 2013;66(6):660-5.

86. Wakimoto DK. Google Scholar Retrieves Twice as Many Relevant Citations as PubMed and Provides Greater Full-Text Access for Quick, Clinical Nephrology Searches. Evidence Based Library & Information Practice. 2014;9(1):36-8.

87. Whiting P, Westwood M, Beynon R, Burke M, Sterne JA, Glanville J. Inclusion of methodological filters in searches for diagnostic test accuracy studies misses relevant studies. Journal of Clinical Epidemiology. 2011;64(6):602-7.

88. Wieland S, Dickersin K. Selective exposure reporting and Medline indexing limited the search sensitivity for observational studies of the adverse effects of oral contraceptives. Journal of Clinical Epidemiology. 2005;58(6):560-7.

89. Wilson LM, Sharma R, Dy SM, Waldfogel JM, Robinson KA. Searching ClinicalTrials.gov did not change the conclusions of a systematic review. Journal of Clinical Epidemiology. 2017;90:127-35.

90. Wong SS, Wilczynski NL, Haynes RB. Optimal CINAHL search strategies for identifying therapy studies and review articles. J Nurs Scholarsh. 2006;38(2):194-9.

91. Wright K, Golder S, Rodriguez-Lopez R. Citation searching: a systematic review case study of multiple risk behaviour interventions. BMC Med Res Methodol. 2014;14:73.

92. Wu YP, Aylward BS, Roberts MC, Evans SC. Searching the scientific literature: implications for quantitative and qualitative reviews. Clin Psychol Rev. 2012;32(6):553-7.

93. Zheng MH, Zhang X, Ye Q, Chen YP. Searching additional databases except pubmed are necessary for a systematic review. Stroke. 2008;39(8):e139.

94. Zwakman M, Verberne LM, Kars MC, Hooft L, van Delden JJM, Spijker R. Introducing PALETTE: an iterative method for conducting a literature search for a review in palliative care. BMC Palliat Care. 2018;17(1):82.

**Wrong outcome**

1. Affengruber L, Wagner G, Waffenschmidt S, Lhachimi SK, Nussbaumer-Streit B, Thaler K, et al. Combining abbreviated literature searches with single-reviewer screening: three case studies of rapid reviews. Syst Rev. 20

**There are no sources in the current document.**20;9(1):162.

2. Cooper M, Ungar W, Zlotkin S. An assessment of inter-rater agreement of the literature filtering process in the development of evidence-based dietary guidelines. Public health nutrition. 2006;9(4):494-500.

3. Doust J, Pietrzak E, Sanders S, Glasziou P. Identifying studies for systematic reviews of diagnostic tests was difficult due to the poor sensitivity and precision of methodologic filters and the lack of information in the abstract. Journal of clinical epidemiology. 2005;58(5):444-9.

4. Edwards P, Clarke M, DiGuiseppi C, Pratap S, Roberts I, Wentz R. Identification of randomized controlled trials in systematic reviews: accuracy and reliability of screening records. Statistics in medicine. 2002;21(11):1635-40.

5. Gartlehner G, Affengruber L, Titscher V, Noel-Storr A, Dooley G, Ballarini N, et al. Single-reviewer abstract screening missed 13 percent of relevant studies: a crowd-based, randomized controlled trial. Journal of clinical epidemiology. 2020;121:20-8.

6. Gartlehner G, Wagner G, Lux L, Affengruber L, Dobrescu A, Kaminski-Hartenthaler A, et al. Assessing the accuracy of machine-assisted abstract screening with DistillerAI: a user study. Systematic reviews. 2019;8(1):1-10.

7. Gates A, Gates M, DaRosa D, Elliott SA, Pillay J, Rahman S, et al. Decoding semi-automated title-abstract screening: findings from a convenience sample of reviews. Systematic reviews. 2020;9(1):1-12.

8. Gates A, Gates M, Sebastianski M, Guitard S, Elliott SA, Hartling L. The semi-automation of title and abstract screening: a retrospective exploration of ways to leverage Abstrackr’s relevance predictions in systematic and rapid reviews. BMC medical research methodology. 2020;20:1-9.

9. Mortensen ML, Adam GP, Trikalinos TA, Kraska T, Wallace BC. An exploration of crowdsourcing citation screening for systematic reviews. Research synthesis methods. 2017;8(3):366-86.

10. Nama N, Sampson M, Barrowman N, Sandarage R, Menon K, Macartney G, et al. Crowdsourcing the Citation Screening Process for Systematic Reviews: Validation Study. Journal of medical Internet research. 2019;21(4):e12953-e.

11. Pham MT, Waddell L, Rajić A, Sargeant JM, Papadopoulos A, McEwen SA. Implications of applying methodological shortcuts to expedite systematic reviews: three case studies using systematic reviews from agri‐food public health. Research synthesis methods. 2016;7(4):433-46.

12. Rosen L, Suhami R. The art and science of study identification: a comparative analysis of two systematic reviews. BMC medical research methodology. 2016;16(1):1-13.

**Wrong concept**

1. Haddaway NR, Collins AM, Coughlin D, Kirk S. The Role of Google Scholar in Evidence Reviews and Its Applicability to Grey Literature Searching. PLoS ONE. 2015;10(9):e0138237.

2. Ng L, Pitt V, Huckvale K, Clavisi O, Turner T, Gruen R, et al. Title and Abstract Screening and Evaluation in Systematic Reviews (TASER): a pilot randomised controlled trial of title and abstract screening by medical students. Syst Rev. 2014;3:121.

3. Rucker G, Reiser V, Motschall E, Binder H, Meerpohl JJ, Antes G, et al. Boosting qualifies capture-recapture methods for estimating the comprehensiveness of literature searches for systematic reviews. J Clin Epidemiol. 2011;64(12):1364-72.

4. Shemilt I, Khan N, Park S, Thomas J. Use of cost-effectiveness analysis to compare the efficiency of study identification methods in systematic reviews. Systematic Reviews. 2016;5(1):140.

5. Tetzlaff J, Murad MH, Wang Z. AI4 CAN WE DECREASE THE SCREENING BURDEN IN SYSTEMATIC REVIEWS? PERFORMANCE OF TWO NATURAL LANGUAGE PROCESSORS TO EXCLUDE RECORDS. Value in Health. 2020;23:S1-S2.

**Wrong publication type**

1. Affengruber L, Wagner G, Gartlehner G. An ultra-rapid review approach: An innovative solution to make evidence more relevant for time-sensitive policy-making and providing guidance for health systems? BMJ Evidence-Based Medicine. 2019;24:A23.

2. Bell M. Systematic searching?...well, we had a bit of a look. JBI Database of Systematic Reviews and Implementation Reports. 2018;16(2):258-9.

3. Janssens A, Gwinn M. Novel citation-based search method for scientific literature: application to meta-analyses (vol 15, 84, 2015). BMC Med Res Methodol. 2015;15:1.

**Wrong study design**

1. Hirt J, Nordhausen T, Appenzeller-Herzog C, Ewald H. Using citation tracking for systematic literature searching-study protocol for a scoping review of methodological studies and an expert survey. F1000Research. 2020;9(1386):1386.

2. Sanfilippo F, Tigano S, Palumbo GJ, Astuto M, Murabito P. Importance of inclusion criteria in systematic reviews. Br J Anaesth. 2020;125(5):e398-e9.

3. Waffenschmidt S, Hausner E, Sieben W, Jaschinski T, Knelangen M, Overesch I. Effective study selection using text mining or a single-screening approach: a study protocol. Syst. 2018;7(1):166.

**Full text not retrievable**

1. Abe S. Searching the evidence via PubMed. Online Kensaku. 2012;33(3/4):83-109.

2. Karia R, Zwaferink H, Malik MU. Relevance of conference abstracts and hand-searching in systematic reviews. Value in Health. 2009;12(3):A25.

3. Shaw B, Worthington H. Hand-searching for systematic reviews. British dental journal. 2001;190(12):632.
