# Supplementary material for: Characteristics and recovery methods of studies falsely excluded during literature screening—a systematic review
Source: Syst Rev. 2022 Nov 9;11:236. doi: 10.1186/s13643-022-02109-w (PMC9644550; doi:10.1186/s13643-022-02109-w)

**Additional file 5.** Risk of bias for methods studies with case study design based on the Joanna Briggs Institute Critical Appraisal Checklist for Case Reports (14)


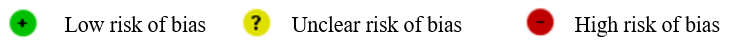

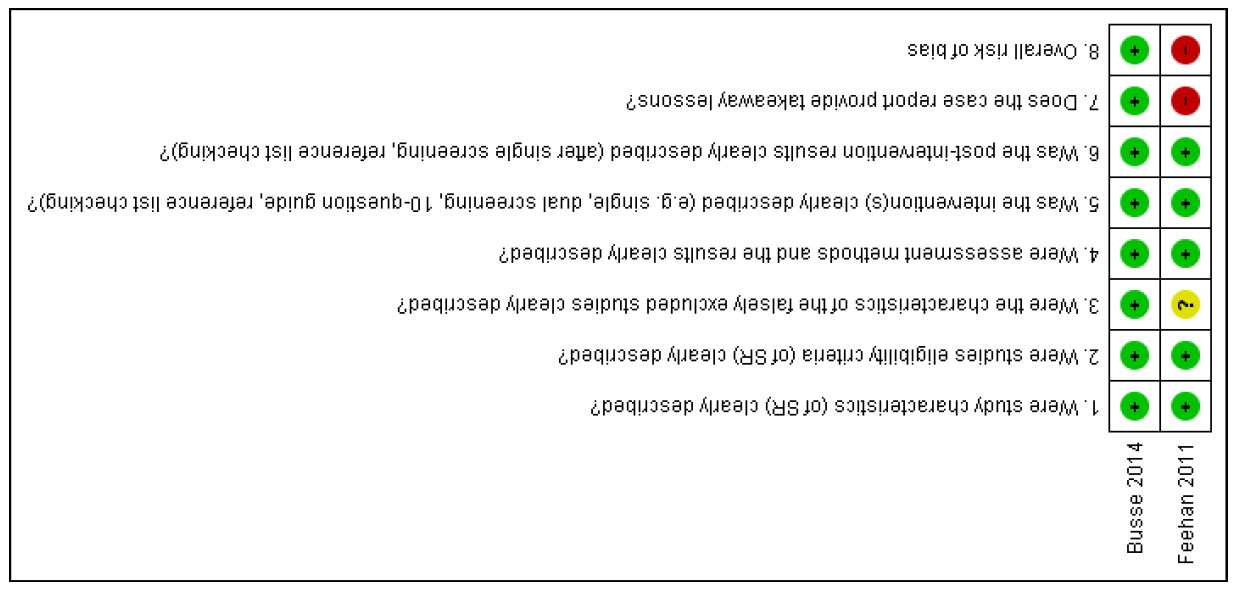


Risk of bias for methods studies with a case series design based on the Joanna Briggs Institute Critical Appraisal Checklist for Case Series (14)


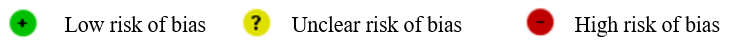

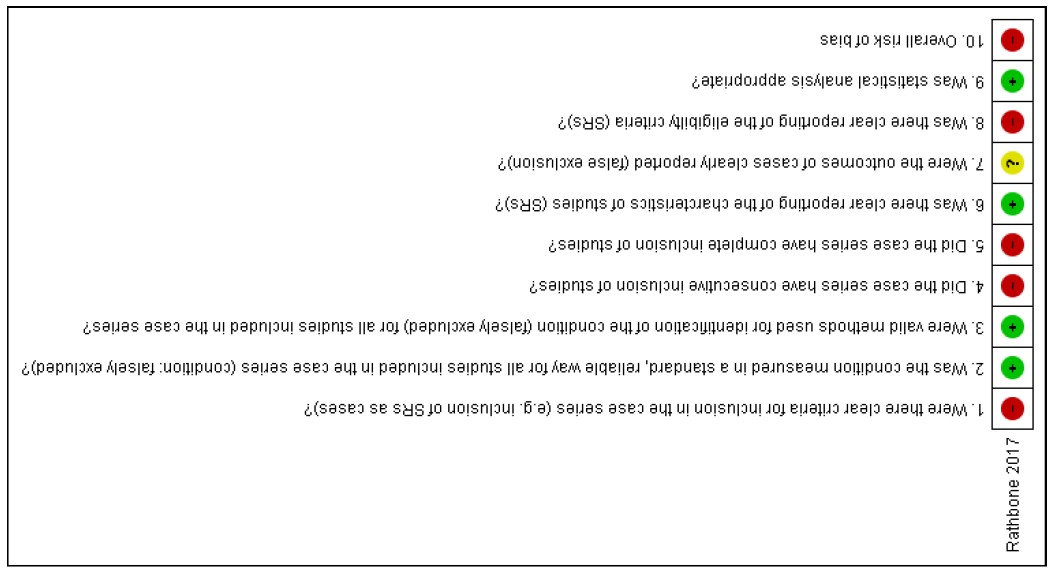

Supplement: Supplementary file 5 — Additional file 5. Risk of bias of included studies. [file 13643_2022_2109_MOESM5_ESM.docx]
